# Effect of online problem-solving counseling on the sexual anxiety and intimacy of women with recurrent pregnancy loss: A clinical trial

**DOI:** 10.18502/ijrm.v22i9.17476

**Published:** 2024-11-14

**Authors:** Shahla Mohammadkhani, Nasrin Ghasemi, Tayebeh Mokhtari Sorkhani, Mahshid Bokaie

**Affiliations:** ^1^Student Research Committee, Shahid Sadoughi University of Medical Sciences, Yazd, Iran.; ^2^Abortion Research Center, Reproductive Sciences Institute, Shahid Sadoughi University of Medical Sciences, Yazd, Iran.; ^3^Department of Health Education and Health Promotion, Social Determinant of Health Research Center, School of Public Health, Shahid Sadoughi University of Medical Sciences, Yazd, Iran.; ^4^Research Center for Nursing and Midwifery Care, Non-communicable Diseases Research Institute, Department of Midwifery, School of Nursing and Midwifery, Shahid Sadoughi University of Medical Sciences, Yazd, Iran.

**Keywords:** Habitual miscarriage, Counseling, Problem-solving, Sexuality, Anxiety.

## Abstract

**Background:**

Recurrent pregnancy loss (RPL) creates complex reproductive conditions among women. Problem-solving therapy is one of the sexual health approaches.

**Objective:**

This study was designed to investigate the effect of online problem-solving counseling on the sexual anxiety and intimacy of women with RPL.

**Materials and Methods:**

A randomized clinical trial was conducted at Abortion Research Center in Yazd, Iran between March and August 2023. A total of 70 women with RPL were assigned into 2 groups, that is, intervention and control using random allocation software (n = 35/each). The intervention group received 8 sessions of sexual counseling on problem solving. The control group received an educational pamphlet. The primary outcome was sexual anxiety, and the secondary outcome was sexual intimacy. The data were collected using questionnaires based on sexual anxiety and intimacy. The questionnaires were completed before, after, and 1 month after the study.

**Results:**

A total of 70 participants were included in the final analysis. The mean score of sexual anxiety in the 8 ${\;}^{\text{th}}$ and 12 ${\;}^{\text{th}}$ wk was significantly less in the online group than in control group (p < 0.001). The mean score of sexual intimacy in the 8 ${\;}^{\text{th}}$ and 12 ${\;}^{\text{th}}$ wk was significantly higher in the online group than in control group (p < 0.001).

**Conclusion:**

Problem-solving-based sexual health counseling programs may improve sexual anxiety and intimacy in women with RPL. It is recommended to use a sexual health counseling method in RPL centers when considering the effectiveness of this type of training.

## 1. Introduction

Recurrent pregnancy loss (RPL) is defined as the failure of at least 2 clinically recognized pregnancies before 20–24 gestation weeks. The American Association of Obstetricians and Gynecologists report that approximately 65% of women with unexplained RPL subsequently conceive. The majority of RPLs (approximately 60%) occur in random outbreaks, and the prevalence of RPL among couples is approximately 1% (1).

Miscarriage is described as a severe sexual crisis for couples (2, 3). Sexual anxiety is a concern about sex that leads to various sexual disorders. Sexual performance anxiety affects 9–25% of men, leading to premature ejaculation and erectile dysfunction. It also affects 6–16% of women, inhibiting their libido. Studies have shown that women with anxiety disorders report poorer sexual performance, and more sexual inhibition than women without anxiety disorders (4, 5). One of the things that is effective in improving the sexual performance of people is sexual intimacy. Sexual intimacy occurs when spouses can easily share feelings and desires emotionally, intellectually, spiritually, sexually, physically, aesthetically, and socially (6, 7). Studies have shown that decreased sexual performance, lack of sexual satisfaction, reduction in sexual relations, common misconceptions, and lack of correct knowledge are common in couples with RPL. They lead to differences between couples and a decrease in mental health, sexual performance, and marital satisfaction (8, 9). The rate of divorce and marital dissolution was significantly higher in the group of women with recurrent miscarriages than in the group of women without recurrent miscarriages (8).

The problem-solving approach involves a cognitive-behavioral process that offers various alternatives for managing problematic situations and increases the likelihood of selecting the most effective response. This method is useful for both individual and group treatments. This text describes a cognitive-behavioral therapy approach that may enhance an individual's ability to manage stressful life experiences. The treatment involves active collaboration between the patient and therapist. Problem-solving treatment is a brief, structured psychological intervention that, like other cognitive-behavioral treatments, focuses on the present rather than past experiences and regrets. The therapy requires the patient and therapist to work together actively, with the patient taking on a more involved role in treatment planning and carrying out activities in between sessions (10).

Due to the lack of sufficient and comprehensive studies around sexual health interventions for couples with RPLs, this study aimed to investigate the effect of online problem-solving counseling on sexual anxiety and intimacy in women with RPL. This article is published with code https://doi.org/10.21203/rs.3.rs-3584682/v1 on the preprint site for introduction.

## 2. Materials and Methods

### Study setting

This interventional parallel randomized clinical trial was conducted between March and August 2023 using a before-after design with a control group and a 1-month follow-up. The statistical population of this research were all women with RPL who were referred to the Abortion Research Center, Yazd Reproductive Sciences Institute, Yazd, Iran for treatment. Eligible participants were contacted based on the center's list according to inclusion criteria.

### Sample size

Sampling was done based on the inclusion criteria by examining the available documentation. The required sample was determined in each group using the following formula. According to a study, the sample size was calculated to be 70 (n = 35/ each) (11). In this formula, 1- α and 1- β are the reliability and test power levels, respectively, and were considered equal to 0.95 and 0.80. As a result, Z_ (1- α /2) and Z_ (1- β ) of the normal distribution table were 1.98 and 0.58, respectively. 


$$n=\frac{2{S_{p}}^{2}{\left[Z_{1-\alpha /2}+Z_{1-\beta }\right]}^{2}}{{\mu_{d}}^{2}}$$

### Randomization, blinding, and concealment

The first-generation random allocation method was used to assign the eligible participants into 2 intervention and control groups, each participant was given a number from 1–70. Then, using the website http://www.randomization.com, participants were randomly assigned into 2 blocks of 35, C, and D. For concealment, a person external to the study used a random lottery method to select each block as an intervention or control group. Blinding was not done because the participants were aware of the sexual counseling sessions.

### Participants

This randomized clinical trial included 70 women with a history of at least 2 repeated miscarriages, aged between 18 and 45 yr, no history of major stress such as death of relatives in the last 3 months, access to social network and a smartphone, and being Iranian.

Exclusion criteria are the patient's or her spouse's affliction with serious psychological disorders such as severe depression and psychosis, consumption of any drug affecting sexual relations by the patient or her spouse (self-reported), history of participating in sexual health education and counseling sessions, and pregnancy.

### Intervention

To conduct the research, the participants provided their phone numbers to participate in the study. The time and date of the counseling sessions were announced by phone. Informed consent was obtained from the participants by creating an online informed consent form. After obtaining consent and explaining the research objectives, demographic information forms and sexual anxiety and intimacy questionnaires were also completed by all the participants before the intervention (baseline), after the intervention (8 ${\;}^{\text{th}}$ wk), and during the 4 wk follow-up (12 ${\;}^{\text{th}}$ wk).

The first author held sexual counseling program sessions with a problem-solving approach for the intervention group. She had a certificate of ability to perform a problem-solving approach under the supervision of the 4 ${\;}^{\text{th}}$ author. This counseling session was conducted based on previous related studies and opinions of experts, including a Ph.D. in sexual and reproductive health.

The content of the sessions is programmed in table I. Weekly, 8 sessions (90 min) of problem-solving counseling were held for the intervention group (10). The sessions were conducted online using the social network, and an educational pamphlet on problem-solving via social networking was sent to control group. The questionnaire was filled out by participants under the supervision of an M.Sc. student.

### Study outcomes and variables

The primary outcome of the present study was sexual anxiety, and the secondary outcome was sexual intimacy. Study variables were collected from demographic information forms and sexual anxiety and intimacy questionnaires. We categorized study variables into 2 groups: a) baseline variables, including demographic characteristics patient's age, spouse's age, patient's job, spouse's job status, patient education, spouse's education, and information on RPL including the number of miscarriages and the duration of the last miscarriage. b) outcome variables including sexual anxiety and sexual intimacy.

#### Sexual anxiety questionnaire

The questionnaire consisted of 18 questions, with a Likert scale from 1–5. This questionnaire had acceptable validity in Iran. Cronbach's alpha for all items was above 0.75. Confirmatory factor analysis established the final factor construct of this questionnaire (12).

#### Sexual intimacy questionnaire

The questionnaire consisted of 5 questions, with a Likert scale from 1–10. This scale is the sexual part of the marital intimacy of Bagarrozie questionnaire. This questionnaire had acceptable validity in Iran. Cronbach's alpha for all items was above 0.70. Confirmatory factor analysis established the final factor construct of this questionnaire (13).

**Table 1:** Details of the content presented in the sessions with a problem-solving approach

**Session**	**Session goal**	**Session content**	**Home practice**
**1**	- Getting to know - Statement of rules - Vision	The introduction, anatomy, and physiology of the reproductive system, the sexual cycle, the female genitalia, the erogenous zones, and factors affecting them, an explanation of how to fill in the questionnaire, and presenting home practice	- Making a list of sexual anxieties to work on - Recording these problems
**2**	Targeting	The importance of sexual relations and the lack of concentration on penetration in all sexual relations of the couple, discussing RPL and its effects, treatments, and complications	- Understanding and evaluating the causes of sexual anxiety - Prioritizing these subjects
**3**	- Discovery - Exceptions	Training in problem-solving skills, coping with stress, relaxation techniques, effective communication, and discussing alternative solutions to improve the couple's relationship when experiencing RPL	Prepare a list of your negative thoughts related to RPL
**4**	Highlighting abilities	The review of sexual schemas and RPL, and their control, review of their sexual expectations, training sexual skills, sensational exercises, and snowball technique	Practicing the snowball technique and discovering emotion-oriented and problem-oriented techniques
**5**	Dominance and power	Free discussion regarding issues and problems, defining exact conformation of the issue, determining a range of possible solutions, analyzing solutions, problems created following the execution of individual solutions, weaknesses, and strengths of individual solutions, anticipating consequences, developing and strengthening communication skills, managing negative excitement (negative anger and excitement), training relaxation, and teaching creative visualization techniques	Challenging beliefs of their post-sexual anxiety and practicing creative visualization techniques
**6**	Feedback and admiration	Providing suitable solutions against complications caused by RPL in sexual matters, changes in appearance, improving communication techniques, reducing worries through counseling, introducing the Kegel exercise, and other effective factors in strengthening sexual anxiety (nutrition, concentration on moments of enjoying it)	Examining positive and negative solutions in solving problems
**7**	A celebration of change	Examining the task of the sixth session. Implementing the best solution chosen by the members. Thanks for the changes	Implementing the best solution
**8**	Conclusion	Reviewing the development and setting the follow-up meeting. Completion of questionnaires	Preparation of the development until the follow-up period
RPL: Recurrent pregnancy loss			

### Ethical Considerations

This study was conducted based on the Declaration of Helsinki and the approval of the Ethics Committee of the Shahid Sadoughi University of Medical Sciences, Yazd, Iran (Code: IR.SSU.REC.1401.078). Additionally, after explaining the objectives of the study to the participants, informed written consent was obtained from all the participants, and they were assured of the confidentiality of the study results. This clinical trial has been registered in the Iranian Registry of Clinical Trials on February 14, 2023 and updated on April 10, 2024.

### Statistical Analysis

Statistical analysis of the data extracted from the questionnaires was done using SPSS 16 software (Statistical Package for Social Sciences version 16.0, Chicago, Illinois, USA). Descriptive statistics were used to describe data, draw tables, calculate percentages, mean, standard deviations, and inferential statistics were used to compare the mean of variables in each group. The Chi-square test was used to compare the qualitative variable. One sample Kolmogorov-Smirnov test was used to check the normality of the distribution of the study data.

Due to the normality of the distribution of the quantitative variables related to the demographic questionnaire, the parametric independent sample *t* test was used to compare the variables in the 2 groups. Also, due to the normality of the data distribution, repeated measurement ANOVA was used to compare within the group, the mean score of sexual anxiety and intimacy 3 times for each group (p < 0.05).

## 3. Results

**Figure 1:**
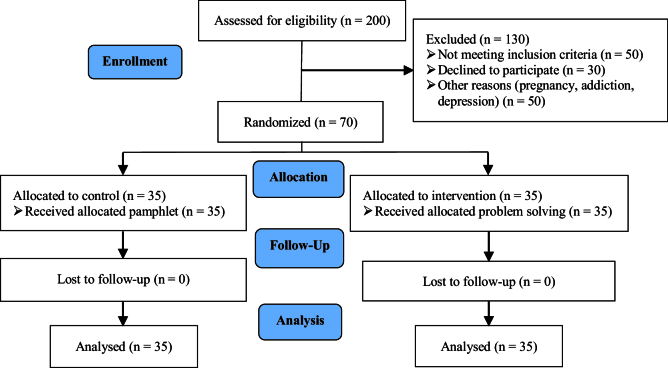
CONSORT flow diagram.

Initially, 200 women with RPL were enrolled in the study. A total of 130 participants were excluded due to not meeting the inclusion criteria (50 cases), declining to participate (30 cases), and other reasons (50 cases). The remaining participants were randomly assigned to the intervention and control groups (n = 35/each). Finally, the data of 70 people (n = 35/each) were statistically analyzed (Figure 1). No significant difference was observed between the groups in terms of demographic characteristics (p ≥ 0.05) (Table II).

Also, the score of sexual anxiety was significantly different 3 times between the control and the intervention groups. Sexual anxiety in the intervention group was obtained at 62.8 ± 10.71, 53.17 ± 7.13, and 49.74 ± 7.09, and in the control group 62.00 ± 6.43, 61.34 ± 6.13, and 61.25 ± 5.96 before, after, and one month after the intervention, respectively (Table III). The statistical test shows the difference in means of sexual anxiety in the 2 groups. Sexual anxiety before and immediately after the intervention was significantly different (p < 0.001), before and one month after the intervention was significantly different (p < 0.001), and also immediately after the intervention and one month later was significantly different (p < 0.001). No significant difference was observed in the control group at any time. Sexual intimacy was not significantly different before the intervention (p = 0.576), but immediately after the intervention (p < 0.001) and one month after the intervention (p < 0.001) was significantly different (Table IV). Sexual intimacy in the intervention group before and immediately after the intervention was significantly different (p < 0.001), before and one month after the intervention was significantly different (p < 0.001), also immediately after the intervention and one month later was significantly different (p < 0.001) (Table IV).

**Table 2:** Demographic characteristics of the studied samples in 2 groups

**Variable**		**Intervention**	**Control**	**95% CI**	P-value*
Age*		29.40 ± 5.00	29.82 ± 5.31	-2.89–2.03	0.73
Spouse's age*		31.57 ± 5.64	31.57 ± 5.74	-2.71–2.71	1
Number of miscarriages*		3.77 ± 1.64	3.65 ± 1.65	-0.66–0.89	0.77
Duration of last miscarriage*		12.82 ± 7.20	12.22 ± 7.53	-2.91–4.11	0.73
Education**					
	**Under diploma**	15 (42.9)	16 (45.7)		
	**Diploma**	14 (40)	14 (40)		
	**Academic**	6 (17.1)	5 (14.3)		0.94
Spouse's education**					
	**Under diploma**	13 (37.1)	14 (40)				
	**Diploma**	12 (34.3)	9 (25.7)		
	**Academic**	10 (28.6)	12 (34.3)		0.72
Spouse's job***					
	**Freelance**	6 (8.6)	4 (5.7)				
	**Employee**	11 (15.7)	11 (15.7)		
	**Manual**	18 (25.7)	20 (28.6)		0.80
Job***					
	**Housewife**	18 (51.4)	22 (62.9)				
	**Employed**	17 (48.6)	13 (37.1)		0.46
*Data presented as Means ± SD, Independent sample *t* test. **Data presented as n (%), Chi-square test. ***Fisher's exact test. CI: Confidence interval					

**Table 3:** Comparison of sexual anxiety variable in 3 times in 2 groups

**Sexual anxiety**	**Intervention**	**Control**	P-value*	P-value**
**Before intervention**	62.8 ± 10.71	62.00 ± 6.43	0.706	
**Immediately after the intervention**	53.17 ± 7.13	61.34 ± 6.13	< 0.001	
**One month after the intervention**	49.74 ± 7.09	61.25 ± 5.96	< 0.001	
**Time 0–8**	** < 0.001	** < 0.001	-	
**Time 8–12**	** < 0.001	** < 0.001	-	
**Time 0–12**	** < 0.001	** < 0.001	-	< 0.001
**P-value between group time**	* < 0.001		-		-
Data presented as Mean ± SD, *Independent *t* test (between groups), **Repeated measure ANOVA (before-after)				

**Table 4:** Comparison of sexual intimacy variable in 3 times in 2 groups

**Sexual intimacy**	**Intervention**	**Control**	P-value*	P-value**
**Before intervention**	28.68 ± 2.75	28.31 ± 2.77	0.576	
**Immediately after the intervention**	32.05 ± 2.22	28.31 ± 2.60	< 0.001	
**One month after the intervention**	33.25 ± 2.35	29.31 ± 2.59	< 0.001	
**Time 0–8**	** < 0.001	**1	-	
**Time 8–12**	** < 0.001	** < 0.001	-	
**Time 0–12**	** < 0.001	**0.021	-	< 0.001
**P-value between group time**	* < 0.001		-		-
Data presented as Mean ± SD, *Independent *t* test (between groups), **Repeated measure ANOVA (before-after)				

## 4. Discussion

The main goal of this study was to investigate the effectiveness of online counseling with a problem-solving approach in the sexual anxiety and intimacy of women with RPL. Psychological management for healthcare providers as well as patient involvement in management would recover the patient experience with spontaneous miscarriage care. In this systematic review study, the importance of psychological counseling of women after repeated abortions by health workers was discussed, and the present study was conducted in line with the recommendation of some researchers to conduct counseling interventions on women with repeated abortions (14).

In a study, the links between sexual anxiety sensitivity and sexual well-being were discussed. Anxiety sensitivity, the fear of feelings of sexual arousal, is related to poorer sexual performance, greater sexual anxiety, and less sexual frequency (15). In a study in Khorramabad, it was shown that relaxation techniques could reduce sexual anxiety in females with infertility (16). The results of this study are consistent with the results of the current study. In a study conducted on 46 women who had a spontaneous miscarriage in their first pregnancy found that 8 sessions of 90 min problem-solving group counseling can be effective in improving mental health after intervention (17). The present study was consistent with this study and similar in terms of time required to complete the questionnaires and the type and number of intervention sessions. The problem-solving approach with a positive orientation leads couples to find effective solutions to their problems and issues and enjoy their married life more. In our study, we focused on 2 variables: sexual anxiety and intimacy.

We found no study on sexual anxiety with a problem-solving approach in women with recurrent miscarriages. But many studies show the positiveness of this approach such as people's abilities and sexual performance (18, 19). A research study investigated the mediating role of 3 variables (sexual awareness, sexual anxiety, and sexual self-esteem) in the path analysis model and showed that there is a relationship between insecure attachment and sexual anxiety (20). The results of a study in Iran showed that schemas mediate the sexual performance of women. This means that if a person's sexual level is high, the treatment alone can temporarily improve sexual function. Studies have recommended interventional studies in this field (21).

The second consequence was the investigation of the sexual intimacy of these women. Problem-oriented coping strategy increased sexual intimacy in women with multiple sclerosis (22). In a study in Iran, a solution-oriented approach improved the sexual satisfaction of women with a high body mass index (23). Solution-oriented couple therapy was effective in improving the sexual intimacy of women with sexual conflicts. The follow-up period in this study was one month (24). The authors emphasized the use of psychological approaches to reduce conflicts and increase sexual intimacy, which was in line with the present study. In agreement with choosing the online method for the intervention, the present intervention was in line with the study below.

Online group counseling with the possibility of private chat may be a better answer to improve sexual performance in a safe, accessible, and effective way (25). Also, research has shown that online cognitive-behavioral interventions are effective for general and social anxiety, reducing depression, and retention in treatment (26). One study showed that both in-person and telephone sexual health counseling improved sexual functioning in breastfeeding women (27). In their clinical study using a remote emotional therapy approach and through online tools, it was stated that online training is an excellent way to provide psychological services (28). Our study was consistent with these studies and showed that remote and online counseling is an effective and feasible approach.

In a study it was showed that the satisfaction of pregnant women was higher with the online method (29). In agreement with choosing the online method for intervention, the present intervention was in line with the above study. The present study differed from their study in the type and duration of the counseling approach. A study showed that the sexual satisfaction of women with infertility was increased after the mindfulness technique. Creating positive psychology may reduce stress and anxiety (30). This study is also in line with our study; however, this research was different from the present study in terms of the type and method of education.

A study demonstrated that a counselor's problem-solving intervention had a moderate effect on improving psychosocial outcomes among adolescents, compared to using problem-solving booklets alone (31). In one study, they showed that both online counseling and pamphlets could improve marital intimacy among mothers of children with Down syndrome, but online counseling was more effective than the other. The use of pamphlets is acceptable for those who could not attend the online meetings (32). The current study found online counseling to be more effective than pamphlets. The intervention method was more effective, as people had homework and consultation with clients in the next session.

### Limitations and strengths 

This study was strengthened by the clients' willingness to participate in counseling sessions and communicate with their counselors if they had medical questions over time. Given the geographical dispersion of study participants across multiple provinces and the limitations on holding in-person meetings, this method proved to be a cost-effective solution for non-native people.

One of the limitations of this study is the presence of cultural issues such as shying away from expressing sexual problems, stress, and concerns related to the process of diagnosis and treatment, which could affect people's responses. To solve this problem of ensuring the confidentiality of information, not needing to mention names, satisfied them to continue participating in the study. Also, blinding was not done and the sample size in the study is small, as a result, it is recommended that this research be conducted with a larger statistical population.

## 5. Conclusion

Sexual health counseling with a problem-solving approach caused a significant decrease in sexual anxiety and a significant increase in sexual intimacy in women with RPL. As a result, it can be said that problem-solving counseling as one of the therapeutic approaches deals with the positive adaptation of people against negative life events. Additionally, this skill helps women define their problems, assess all possible solutions, and select the most appropriate one to effectively deal with their daily life issues. Therefore, considering the many sexual problems of women suffering from recurrent miscarriages, it is suggested that counselors and therapists use this short-term, non-pharmacological, and effective approach in RPL centers.

##  Data Availability

Data supporting the findings of this study are available upon reasonable request from the corresponding author.

##  Author Contributions

N. Ghasemi and M. Bokaie designed the study. Sh. Mohammadkhani conducted, evaluated, and analyzed the results of the study. Further, T. Mokhtari Sorkhani monitored, reviewed and evaluated the result of the study. All authors approved the final manuscript and take responsibility for the integrity of the data.

##  Conflict of Interest

The authors declare that there is no conflict of Interest.
